# Valorization of Uncharred Dry Leaves of *Ficus benjamina* towards Cr (VI) removal from Water: Efficacy Influencing Factors and mechanism

**DOI:** 10.1038/s41598-019-55993-z

**Published:** 2019-12-18

**Authors:** Preeti S. Kulkarni, Varuna S. Watwe, Abubakar J. Hipparge, Sana I. Sayyad, Rutika A. Sonawane, Sunil D. Kulkarni

**Affiliations:** 1 Post-graduate and Research Centre, Department of Chemistry, MES Abasaheb Garware College, Pune, India; 2 Post-graduate and Research Centre, Department of Chemistry, Shikshana Prasarak Mandali’s Sir Parashurambhau College, Tilak Road, Pune, India

**Keywords:** Pollution remediation, Pollution remediation

## Abstract

The potential of uncharred biomaterial derived from dry leaves of *Ficusbenjamina* (Family: Moraceae,local name: Weeping Fig) plant to remove Cr(VI) from aqueous samples was investigated. In the present work, treatment of dilute acids was used for activating the adsorption centres on the biomass instead of cumbersome charring process. The plant material was characterized using FT-IR, FE-SEM and EDX. Various influencing factors such as pH of equilibrating solution, contact time, Cr (VI) concentrations, adsorbent dose and temperature were optimized to obtain maximum sorption efficacy. The interactions among the biomaterial and Cr (VI) in water were studied by fitting the sorption data in four different adsorption isotherms. The data fitting and experimental evidences indicated formation of monolayer of Cr(VI) over the biomass surface. The process followed pseudo-second order kinetics and was thermodynamically spontaneous under laboratory conditions and reached equilibrium in 24 hours. Maximum adsorption capacity of 56.82 mg/g was obtained at the pH 2 when the concentration before adsorption was 200 mg L^−1^ of Cr(VI) with 24 hours of equilibration time and 2.50 g L^−1^ of dose of biomaterial at room temperature. The sorption efficiency was found to be better than many charred bio-based materials.

## Introduction

India is a developing country with rapid growth in industries such as manufacturing, pharmaceuticals, textile, mining and agriculture. The growing industrialization requires a large quantity of water that becomes contaminated at the end of industrial process. This leads to depletion of clean and potable water for living organisms. Contaminated water after treatment flows into the water bodies but still contains some quantities of pollutants that are not removed during the effluent treatment. These contaminants may pose a serious risk to health of living organisms^1,2^.

The most common industrial contaminants are heavy metals in their stable oxidation states. Their salts are soluble and highly mobile in water bodies and hence removing them presents a major challenge. Their removal requires specialized chemical and physical treatment methods which are often costly. Chromium (Cr) is one of the heavy metalsthat poses major threat to human health because it is 16^th^ most toxic element according to the Agency for Toxic Substances and Disease Registry (ATSDR)^3^. The two most common oxidation states of Cr in environment are Cr(III) and Cr(VI). Cr(III) is mostly generated due to natural activities and is implicated to be an essential trace element for humans and helps in glucose tolerance^4^. Cr(VI) is generated mainly due to human anthropological activities like electroplating, leather tanning, cement, and textile industries^5^ and is toxic to living organisms even at trace level^3^. It is reported that Cr(VI) is 500 times more toxic than Cr(III)^6^. The effects of exposure to Cr(VI) cause eye irritation, perforated eardrums, liver damage, upper abdominal pain, respiratory cancer, occupational asthma, pulmonary congestion and discoloration of teeth^3^.

So it becomes imperative to develop environmentally benign methods and eco-friendly materials to remove Cr(VI) from waste waters. There are various methods by which Cr(VI) can be removed from waste water that include chemical precipitation, membrane filtration, reverse osmosis and ion exchange however; these methods are either expensive or less efficient^7,8^. Over these methods bio-sorption serves as an effective strategy as it is cost effective, eco-friendly and logistically easier. It also has other advantages such as metal selectivity, competitive performance, possibility of regeneration and recycling and no sludge generation^9^. Bio-sorption involves a sorption process with fast reversible reaction of pollutants with the biomaterial. Many inexpensive materials have been used for Cr (VI) removal using, corn straw, grape stalks and yohimbe bark, fungus *Arthrinium malaysianum*, pistachio hull waste^10–12^. It is a common practice to char the bio-material to activate adsorption centres on the material surface for efficient removal of target pollutant^13^.

In the present studies we aim to evaluate the efficacy of uncharreddry leaves of *Ficus benjamina* (FB)as an eco-friendly and green sorbent for removal of Cr(VI) from aqueous samples. In India, the plant has common occurrence. In summer each tree generates around 5–6 kg of dry leaves waste per day. There are no reports of its use in removal of Cr species from waste water. Dry leaves of FB were acid treated and characterized using the techniques such as IR, SEM and EDAX. Various parameters such as pH, concentration of Cr, temperature, biomass dose were optimized for maximum Cr removal. The kinetic, thermodynamic and adsorption data obtained were employed to explain the plausible mechanism of Cr (VI) removal by the biomass. Advantage of the method is better adsorption efficiency and reduction in the processing steps during material preparation.

## Results and Discussion

### Acid treatment of FBB

**Figure 1:**
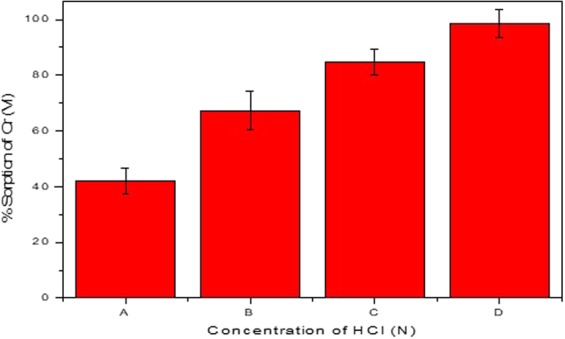
Effect of concentration of acid used for activating the sites on surface of FBB on its sorption capacity. If the concentration of acid used for pre-treatment is greater than 1 mol L^−1^, FBB disintegrates and loses its original physical appearance.

Acid treatment is essential for enhancing the sorption capacity of uncharred bio-adsorbents to activate the adsorption sites like amino, carbonyl, carboxyl, amide and phosphate groups present on its surface^14^. This is due to the fact that under acidic conditions the functional groups on the surface bio-adsorbents get protonated and attract the negatively charged oxyanion species of Cr (VI) present in the aqueous solution^15^. Figure 1 shows the effect of concentration of acid on the sorption capacity of FBB. Untreated FBB showed around 42% of Cr (VI) sorption, however it was observed that the adsorbed Cr (VI) leaches out after 24 h. This might be either due to the weak physio-adsorption of the Cr (VI) on FBB surface or its entrapment in the surface pores making it vulnerable to desorption in due course of equilibration time. Figure 1 clearly illustrates that the percentage sorption of Cr (VI) on FBB increases significantly with the acid concentration, however at very high concentrations of the acid (>1 N) the FBB shows disintegration and changes its physical appearance.

### Characterization of FBB

**Figure 2:**
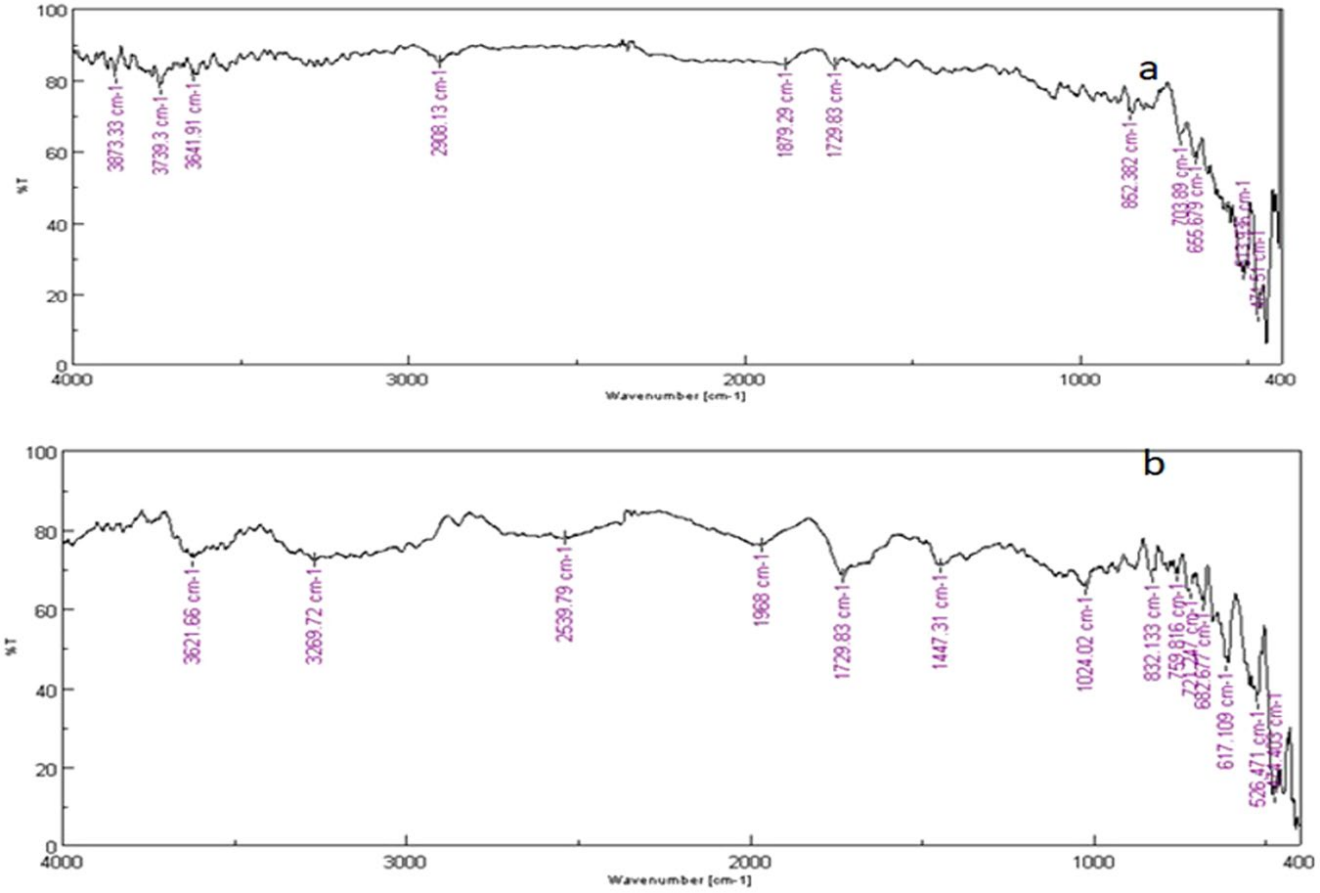
(**a**) IR spectrum of FBB sample before Cr (VI) loading, (**b**) IR spectrum of FBB after Cr (VI) adsorption. The peak positions of many absorption peaks are shifted due to Cr (VI) adsorption.

Figure 2a,b shows the FT-IR spectrum of Cr(VI) loaded and pristine FBB. In pristine FBB Fig. 2a, the adsorption peaks around 3623, 3329, 2920, 2860, 1730, 1602 and 1073 cm^−1^ were due to presence of free hydroxyl group, intermolecular bonded hydroxyl group, C-H group vibrations, carbonyl stretching, C = C stretching and-OCH_3_ groups respectively. It is known that, these groups are generally present on biomaterial^14^. It can also be seen from Fig. 2b, that there is clear shift in the group frequencies due to Cr loading on FBB.

**Figure 3:**
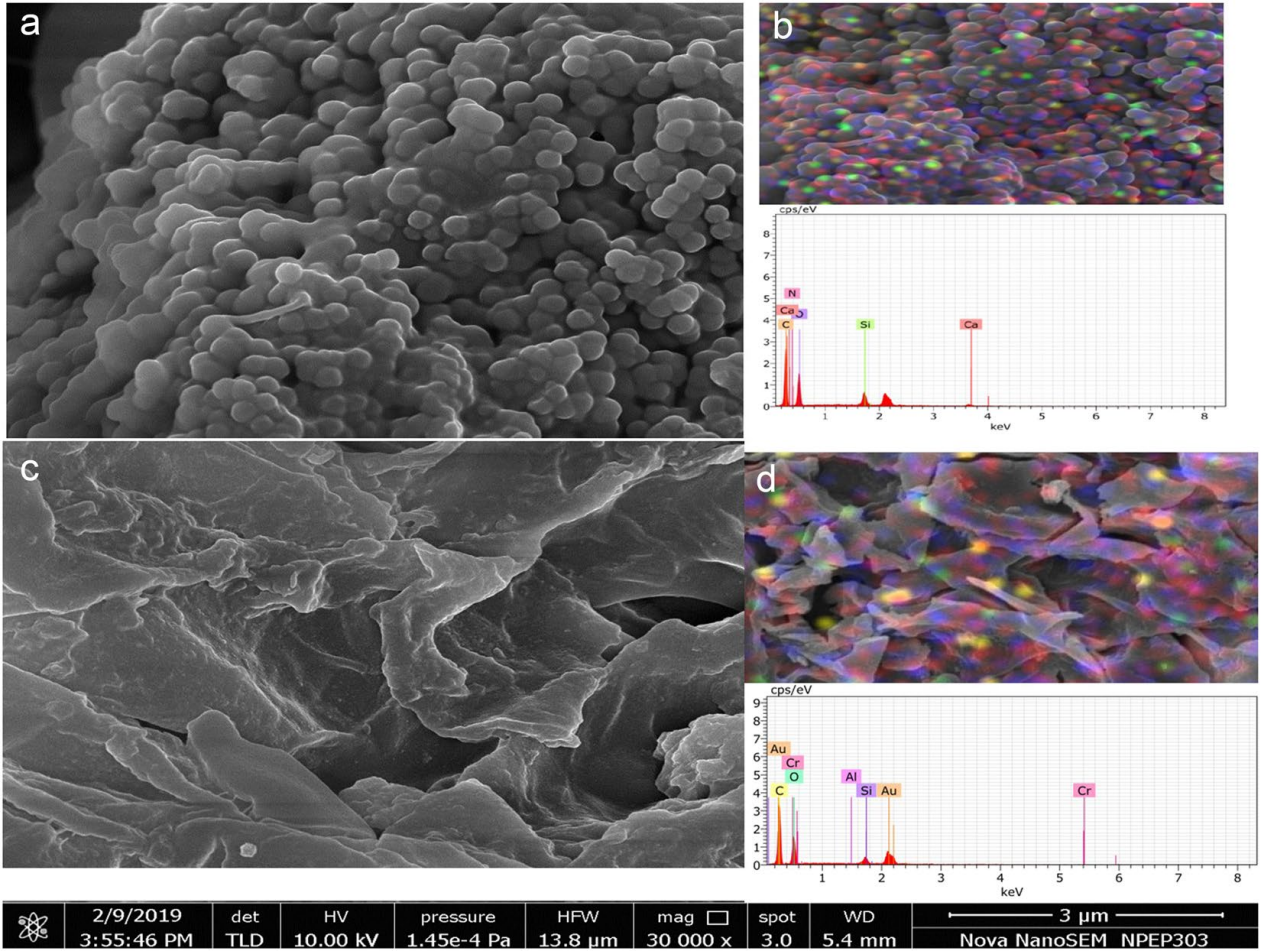
Characterization of FBB using field emission-scanning electron microscope (SEM) and energy dispersive X-ray analysis, (**a**) SEM of FBB before Cr (VI) adsorption, (**b**) qualitative mapping of elements on FBB surface using EDAX (**c**) % elemental composition on FBB surface before Cr (VI) adsorption. Figures (d), (e) and (f) are SEM images, EDAX analysis and elemental composition of FBB after Cr (VI) adsorption.

Figure 3 shows FE-SEM images and EDAX spectrum of pristine and Cr(VI) loaded FBB. The FBB surface shows a layered structure which canfavour metal sorption. The FE-SEM image of the Cr (VI) loaded FBB shows the presence of lustrous species over its surface which are absent in the pristine FBB before metal sorption which are attributed to Cr confirming its presence of on the surface of FBB. The presence of Cr is also confirmed by EDAX spectrum. The % composition of all the elements present on FBB surface is tabulated in Supplementary Table S1.

### Optimization of experimental variables for Cr (VI) sorption

Before testing the efficacy of the FBB prepared in the present work for Cr (VI) sorption, various experimental parameters were optimized.

### pH of equilibrating solution

**Figure 4:**
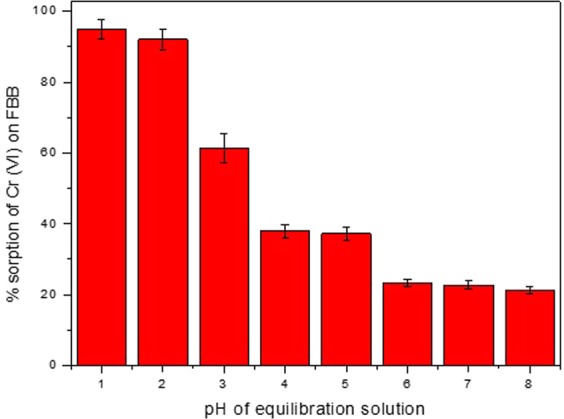
Effect of pH of equilibrating solution of Cr (VI) on sorption capacity of FBB. The pH of 20 mL solution of concentration 100 mg L^−1^was varied in between 1 to 8 using dilute solutions of NaOH and HCl.

Figure 4 shows the % sorption of Cr (VI) on FBB surface at pH values varying in the range of 1 to 8. It was found that % sorption was higher at lower pH values and decreasing successively with increase in solution pH. These results are in concurrence with the point of zero charge which is equal to 4. The sorption studies at pH < 1 of equilibrating solution found to deform FBB. It is known that, the predominant species of Cr(VI) present in the solution at pH values below 2 is HCrO_4_^2−^ ^14^. Under acidic conditions, the FBB undergoes additional protonation thereby providing the driving force required for electrostatic attraction between the oxy-anionic chromate ion in the aqueous solution and the protonated group on the FBB^15^. This synergistic effect of acidic equilibrating solution and protonated FBB is reflected in the high percentage extraction of Cr(VI) at pH value of 1 and 2. At high values of pH due to comparatively less acidic conditions that do not favour protonation, the % sorption was lower^16^.

### Time of equilibration on the uptake of Cr (VI)

**Figure 5:**
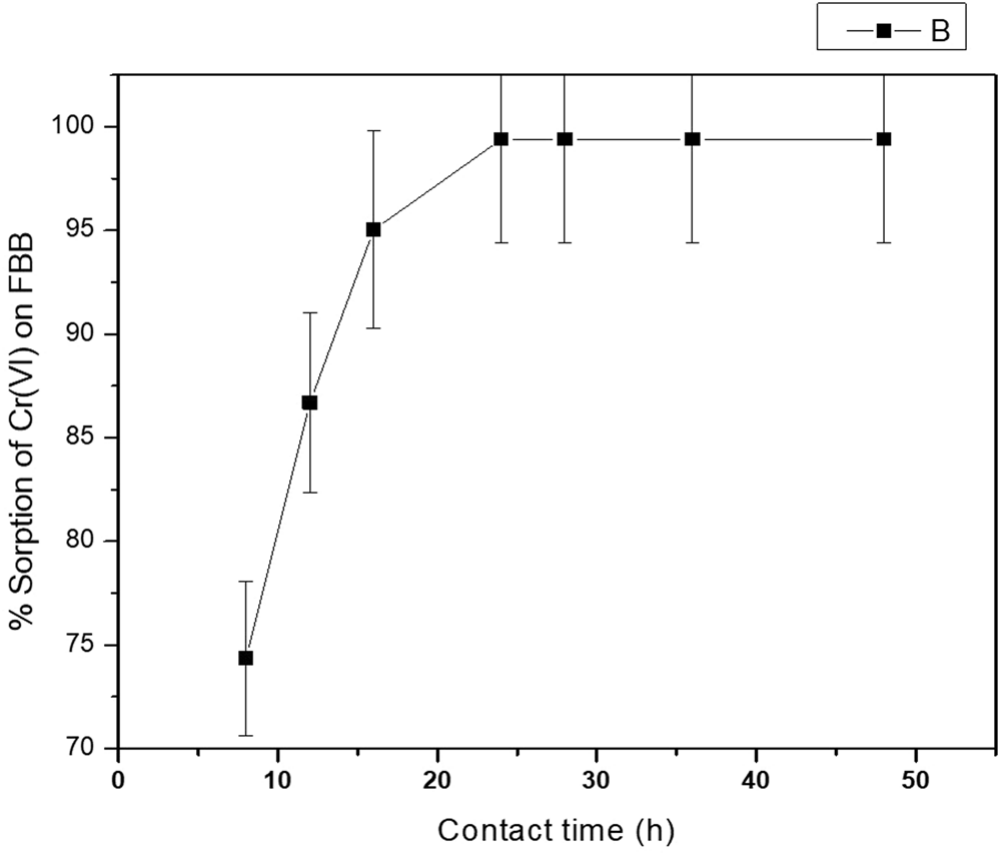
Effect of equilibration time on sorption capacity of FBB 0.01 g of FBB was equilibrated with 20 mL solution of concentration 100 mg L^−1^. The solution was equilibrated for 48 hours and was tested periodically for Cr (VI) at definite time intervals. It was observed that after 24 hours of equilibration, the % sorption almost remains constant.

Figure 5 shows the % Sorption of Cr (VI) as a function of time. It shows that the equilibrium Cr (VI)_aq_⇔Cr (VI)_FBB_, is reached within 24 hours of equilibration time indicating full saturation of adsorption sites on FBB surface. There are many evidences in literature where the biomaterials took time in the range of 1 to 50 hrs to reach the equilibrium^17–22^. This observation propelled us to undertake adsorption kinetic study. The kinetic study of adsorption was carried out to establish the rate of adsorption before the equilibrium reached.

### Adsorption kinetics

There are two methods to study the adsorption-desorption kinetics (1) kinetics study in terms of removal of Cr (VI) from the equilibrating solutions and (2) determination of adsorption orders. Both methods give an important insight of the process. Eq. (15) gives the percentage of Cr (VI) removed from the equilibrating solution as the function of time^23^.

1 $$R=a{(t)}^{b}$$

Where, R is percent removal of Cr(VI), a and b are the constants and t is the contact time in minutes which simplifies to-

2 $$logR=\,\log \,a+b\,\log \,t$$

The plot of % removal of Cr (VI) from the equilibrating solution against time yields the values of constants ‘a’ and ‘b’ Supporting Fig. S2. These values with initial concentrations ranging from 80 to 200 mg L^−1^ are shown in the Table 1. It was observed from values that the ‘a’ values increases from 2.97 for 80 mg L^−1^ to slightly less than thrice of that for 200 mg L^−1^ while that of constant ‘b’ the values decreased from 0.53 to 0.33 in the studied concentration range.

**Table 1:** Kinetic parameters obtained for adsorption of Cr (VI) on FBB.

Initial Cr (VI) concentration ppm	a	B	K_1_ (10^−3^)	R^2^	K_2_ (10^−3^)	R^2^
80	2.97 ± 0.21	0.53 ± 0.04	5.2 ± 0.2	0.7753	2.2 ± 0.1	0.9712
100	3.09 ± 0.28	0.51 ± 0.04	3.8 ± 0.1	0.9012	1.5 ± 0.1	0.9582
150	5.45 ± 0.34	0.40 ± 0.02	3.5 ± 0.1	0.8766	4.3 ± 0.1	0.9932
200	7.13 ± 0.56	0.33 ± 0.02	3.0 ± 0.1	0.8379	4.5 ± 0.1	0.9937

The values ‘a’ and ‘b’ are obtained for removal kinetics using a plot of log R against log t (Eq. 2). k_1_ (min-1) and k_2_ (mg/g min) are the pseudo-first order and pseudo second order rate constants, R^2^ values are the corresponding correlation coefficients. All the values were recorded for triplicate samples and the standard deviation was obtained for a mean of three readings.

The low values of b suggest that with increase in time the rate of percentage removal of Cr(VI) decreases. For lower Cr(VI) concentrations (<80 mg L^−1^) the kinetics was faster and 100 percent removal was obtained in four hours of equilibration time hence data upto 24 hours was not available thus ‘a’ and ‘b’ values were calculated only above 80 mg L^−1^. The observed values of the constants ‘a’ and ‘b’ indicate that for a given dose of biomaterial the uptake capacity is directly proportional to the concentration of Cr(VI) in the equilibrating solution whereas as the adsorption time increases, the second term in Eq. (2) dominates implying at infinite time, the rate of uptake tends to zero as the value of ‘b’ is less than one. This observation can be attributed to the fact that, with the lapse of time, the biomaterial adsorption sites become saturated and no sites are available for further adsorption. The above observation was confirmed by fitting the observed data in pseudo-first order and pseudo-second order reaction kinetics as described in the following section.

The bio-sorption of Cr(VI) by FBB has been tested for pseudo-first order Lagergren equation and pseudo-second order equation shown by Eqs. (3,4) respectively.

3 $$log{q}_{e}-q=log{q}_{e}-(\frac{{k}_{d}}{2.303})t$$

where q_e_ is the mass of metal adsorbed at equilibrium time expressed as mg/g, q is the mass at any other time (min) and k_d_ is the first order reaction rate constant of biosorption (min^−1^) and

4 $$\frac{1}{{q}_{t}}=\frac{1}{2}(K\text{'}{q}_{e}^{2})+\frac{t}{{q}_{e}}$$

Where q_t_ is the mass of metal ion adsorbed at time t (min) and K’ is the pseudo second order rate constant of biosorption (mg g ^−1^min^−1^).

Supporting Fig. S3a,b show the pseudo-first and second order fitting respectively for initial concentrations of Cr (VI) as in the range of 80 to 200 mg L^−1^ for a fixed dose of FBB. Table 1 gives the values of rate constant and corresponding fitting co-relation coefficients. The better fitting was obtained with pseudo-second order kinetics as can be seen from the values of the correlation coefficients. So it can be concluded that, the adsorption follows second order kinetics. Pseudo-first-order kinetic model assumes that the sorption depends only on the number of metal ions present in the solution at a given instant whereas pseudo-second-order model considers both number of adsorption sites on the adsorbent surface in addition to the number of metal ions present in the solution^18,24^, From the kinetics studies, we conclude that, removal of Cr (VI) from the feed solution and its adsorption on FBB moves in the forward direction of the equilibrium and is function of both i.e. the concentration of Cr (VI) in the feed solution as well as the number of adsorption sites on FBB, however after equilibration time of 24 hrs, the adsorption sites become saturated with Cr (VI) and further adsorption is not possible indicating the establishment of the equilibrium between the two participating species.

### Effect of FBB dosage on Cr (VI) uptake

The effect of FBB dosage on metal uptake and percentage removal of Cr(VI) is shown in Table 2. It was observed that the percentage uptake remains almost the same from 0.05 to 0.20 g of FBB/20 ml of equilibrating Cr(VI) solution. Since 0.20 g FBB gives more post process waste, 0.05 g/20 ml was used throughout the study.

**Table 2:** Effect of dose of FBB on adsorption of Cr (VI): The amount of FBB was varied in the range from 0.01 g to 0.2 g keeping the concentration of Cr (VI) constant as 100 mg L^−1^ (20 mL) pH =2. Each dose was repeated thrice and the mean q_e_ has been tabulated with the standard deviation.

Adsorbent dose (g)	q_e_ (mg g^−1^)
0.01	9.02 ± 0.21
0.02	12.22 ± 0.36
0.05	28.41 ± 1.46
0.10	28.65 ± 2.98
0.20	29.48 ± 3.01

### Effect of initial concentration of Cr (VI) in feed solution

The q values calculated using Eq. (16) for the different initial concentrations of Cr(VI) are presented in Table 3. It was observed that the amount of Cr(VI) sorbed gradually increases from 5.14 mg/g for 20 μg mL^−1^ of Cr(VI) to 27.39 mg/g for 80 μg mL^−1^ Cr(VI). This is due to the fact that the efficiency of sorption increases as the ratio of weight of FBB to concentration of Cr(VI) goes on decreasing. However, the q_e_ value shows only an insignificant rise from 27.39 mg/g for 80 μg mL^−1^ to 30.95 mg/g for 200 μg mL^−1^. Thus, the FBB sites available for sorption get saturated for 80 mg L^−1^Cr (VI) solution corresponding to a dosage of 0.05 g/20 ml.

**Table 3:** Effect of initial concentration of Cr (VI) in feed solution on adsorption capacity of FBB: The table shows the calculated q_e_ values for different concentrations of Initial feed solution in the range 20 mg L^−1^ to 200 mg L^−1^ Cr (VI).

Cr(VI) concentration (mg L^−1^)	q_e_ (mg g^−1^)
20	5.14 ± 0.41
40	11.69 ± 1.06
60	13.16 ± 1.36
80	27.39 ± 3
100	28.49 ± 04
150	28.65 ± 2.12
200	30.95 ± 2.89

### Temperature of equilibration bath

Study of effect of temperature enables us to evaluate thermodynamics of adsorption. The values of thermodynamic parameters and percentage of Cr (VI) removed by FBB are presented in Table 4. The data are evaluated at different time intervals (1 to 4 hours) and for four different temperatures in the range from 298.0 to 353.9 K. It is known that high temperature favours adsorption^15^. It can be seen that the percentage extraction of Cr (VI) is 46% at room temperature and increases to approximately 92% at 80 °C. This increase in adsorption at higher temperatures can be attributed to various reasons like increased mobility of metal anion, increased number of adsorption sites, increase in the pore size of the adsorbents, increase in kinetic energy of the metal anion thereby resulting in higher contact time with the adsorbent, increase in diffusion rate across the boundary layer of the adsorbent and also within the adsorbent. The sorption capacity evaluated at different temperatures enabledus to determine the thermodynamic evaluation of the process.

**Table 4:** Thermodynamic data for adsorption of Cr (VI) on FBB surface.

Time (h)	T (K)	∆ G (cal mol^−1^)	∆ S (cal K^−1^mol^−1^)	∆ H (cal mol^−1^)
1	298	1120 ± 46	16.37 ± 1.34	5998 ± 230
	313	875 ± 29		
	333	547 ± 34		
	353	228 ± 19		
2	298	1093 ± 58	25.18 ± 2.87	8596 ± 873
	313	715 ± 56		
	333	211 ± 21		
	353	−(296 ± 23)		
3	298	775 ± 41	27.26 ± 2.11	8895 ± 901
	313	365 ± 11		
	333	−(182 ± 20)		
	353	−(728 ± 30)		
4	298	692 ± 32	27.58 ± 3.01	8912 ± 720
	313	282 ± 56		
	333	−(268 ± 31)		
	353	−(828 ± 29)		
6	298	360 ± 31	36.80 ± 3.12	1127 ± 87
	313	−(186 ± 10)		
	333	−(916 ± 26)		
	353	−(1666 ± 98)		
9	298	−(120 ± 12)	47.60 ± 4	1398 ± 76
	313	−(825 ± 72)		
	333	−(1768 ± 101)		
	353	−(2746 ± 167)		
12	298	−(600 ± 35)	58.40 ± 21	1669 ± 55
	313	−(1460 ± 93)		
	333	−(2620 ± 192)		
	353	−(3830 ± 234)		
16	298	−(1240 ± 101)	72.80 ± 5	2031 ± 105
	313	−(2320 ± 208)		
	333	−(3750 ± 317)		
	353	−(5270 ± 458)		
20	298	−(1880 ± 210)	87.20 ± 67	2392 ± 208
	313	−(3170 ± 294)		
	333	−(4890 ± 518)		
	353	−(6710 ± 467)		
24	298	−(2520 ± 110)	10.16 ± 1.03	2754 ± 267
	313	−(4020 ± 256)		
	333	−(6020 ± 576)		
	353	−(815 ± 78)		

The table summarizes the thermodynamic data obtained for sorption of Cr (VI) on FBB. Different aliquots of 0.05 g FBB was equilibrated with 20 mL of 200 mg L^−1^ Cr (VI) solution for 24 hours at different temperatures using a constant temperature water bath.

For determining thermodynamic parameters, we assumed that, the equilibrium established during the course of adsorption is $Cr\,{(VI)}_{aq}\leftrightharpoons Cr\,{(VI)}_{FBB}$, where Cr (VI)_aq_ is concentration of Cr (VI) in the equilibrating solution and Cr (VI)_FBB_ is its concentration after adsorption with the equilibrium constant K_d_. The value of K_d_ can be determined using Eq. (5) from which other thermodynamic parameters such as ΔG, ΔS and ΔH can be determined by using Eqs. (6,7).

5 $${K}_{d}=\frac{{[Cr(VI)]}_{FBB}}{{[Cr(VI)]}_{ES}}$$

6 $$\mathrm{ln}\,{K}_{d}=\frac{\Delta S}{R}-\frac{\Delta H}{RT}$$

The plot of ln K_d_ against 1/T was a straight line with negative slope. The representative plot is given in the Supplementary Fig. S4. According to Van’t Hoff’s model Eq. (6), the Y-axis intercept and the slope should yield entropy change and the enthalpy of sorption respectively. The values of entropy change and enthalpy were employed to obtain Gibb’s free energy change to predict the sorption spontaneity at different temperatures using the Eq. (7).

7 $$\Delta G=\Delta H-T\Delta S$$

Where T is the solution temperature

It is evident from the data presented in the Table 4 that the Gibbs free energies are positive in the initial stages of equilibration resulting in a non-spontaneous process whereas become negative after three hours of equilibration at higher temperatures. The process is spontaneous for longer equilibration time intervals even at room temperature as observed from the Gibbs free energy values which have decreased from 1.12 Kcal mol^−1^ to 0.692 Kcal mol^−1^ after a period of four hours of equilibration time and become negative after 9 hours of equilibration time. Positive values of ∆H indicate that the sorption of Cr(VI) on FBB is an endothermic process while the positive values of ∆S indicate the movement of Cr (VI) ions from solution to FBB surface during the sorption process.

### Studies on adsorbent-adsorbate interactions

Four different adsorption models viz Langmuir, Freundlich, Dubnin-Radushkevich and Temkin were studied to understand the adsorbate-adsorbent interaction. Equations (8) and (9) represent the expressions for Langmuir and Freundlich model respectively.

8 $${{\rm{q}}}_{{\rm{e}}}=\frac{{\rm{Qmax}}\,{{\rm{K}}}_{{\rm{L}}}\,{\rm{Ce}}}{1+{\rm{KL}}\,{\rm{Ce}}}$$

9 $${{\rm{Q}}}_{{\rm{e}}}={{\rm{K}}}_{{\rm{f}}}{{\rm{C}}}_{{\rm{e}}}^{\frac{1}{{\rm{n}}}}$$

Where Q_max_ is the maximum amount of Cr (VI) adsorbed within a monolayer (mg g^−1^), K_L_ is the Langmuir constant (Lmg^−1^) that depends on Gibbs Free energy of adsorption, K_f_(Lg^−1^) and ‘n’ are the Freundlich adsorption constants. On comparing the Langmuir and Freundlich models it was observed that the Langmuir model showed better fitting as compared to Freundlich model suggesting the adsorption process to be mono-layered with uniform distribution on FBB. Values of n in the range of 2–10 indicates good adsorption characteristics. The separation factors (R_L_) values calculated using following Eq. (10)

10 $${R}_{L}=\frac{1}{1+{K}_{L}{C}_{0}}$$

where K_L_ is Langmuir constant and C_0_ is initial concentration of Cr(VI) in mg L^−1^

For four different initial Cr (VI) concentrations, the R_L_ were showing decreasing trend with increase in concentration. The values were 0.009 and 0.004 respectively for 80 and 200 mg L^−1^ solution of Cr (VI). It is known from the literature that the adsorption process is irreversible if the value of the separation factor is zero, it is favourable if the value is between 0 to 1 and unfavourable if the value is more than one. Further smaller the value of separation factor greater is the affinity between the adsorbent and the adsorbate^17^. According to the above equation the average separation factor calculated for FBB was found to be 0.006 which is appreciably low indicating very strong interaction between FBB and Cr (VI) species.

The expression for Dubinin-Radushkevich model can be represented as:

11 $$lo{g}_{10}{q}_{e}=lo{g}_{10}{q}_{D}-2{B}_{D}^{2}{R}^{2}{T}^{2}lo{g}_{10}(1+\frac{1}{{C}_{e}})$$

Where, q_D_ is the theoretical saturation capacity (mg/g), B_D_ is a constant related to adsorption energy (mol^2^KJ^−2^), R is the gas constant (KJmol^−1^K^−1^) and T is the temperature. The slope and intercept of the curve log_10_q_e_ vs log_10_(1+1/Ce) gives q_D_ and B_D_ values. The energy of adsorption E per molecule (KJ mol^−1^) of adsorbate can be calculated from the constant B_D_ using the following equation^18^.

12 $${E}_{D}=\frac{1}{\sqrt{2{B}_{D}}}$$

The equations for Temkin plots are:

13 $${q}_{e}=B\,ln({A}_{T}{C}_{e})$$

14 $$B=\frac{RT}{b}$$

From the Temkin plot of q_e_ Vs ln C_e_, the slope of the curve gives the Temkin isotherm equilibrium binding constant, A_T_ (L g^−1^) and the intercept gives the Temkin isotherm constant b. Using b value the constant related to heat of sorption B (J mol^−1^) can be calculated. Four adsorption isotherm models viz Langmuir, Freundlich, Temkin and Dubinin-Radushkevich are given in the Supplementary Fig. S5, S8. The constants and the correlation coefficients for all the models are tabulated in Table 5.

**Table 5:** Various constants obtained from adsorption isotherms: The sorption of Cr (VI) on FBB has been evaluated for various adsorption models and the parameters obtained are tabulated.

Parameters	FBB
***Langmuir constants***	
Q_max_ (mg g^−1^)	56.82 ± 4.21
K_L_ (L mg^−1^)	1.408 ± 0.012
R^2^	0.995
***Freundlich constants***	
K_f_(L g^−1^)	27.82 ± 1.27
n (L mg^−1^)	5.46 ± 0.96
R^2^	0.9111
***D-R constants***	
q_D_ (mg g^−1^)	51.629 ± 5.02
B_D_ (mol^2^ KJ^−2^)	0.073 ± 0.002
E_D_ (KJ mol^−1^)	2.62 ± 0.17
R^2^	0.9497
***Temkin constants***	
A (L^−1^ g^−1^)	8.84 ± 0.93
b	263.4 ± 23.8
B(J mol^−1^)	6.37 ± 0.67
R^2^	0.8251

Comparison of sorption capacities of FBB obtained in present studies and comparison with other bio-sorbents as reported in literature gives an idea about potential of FBB for Cr (VI) remedial from waters. Charring is an important step in most of the studies, however we have used uncharred material for Cr (VI) adsorption. In the present studies the maximum Cr(VI) sorption by FBB (q_max_) is found to be 56.82 mg g^−1^ at the optimum conditions and is found to be nearly four times than that of commercial activated charcoal^19–22^. The advantages of using FBB over other materials are that it is economical, easily available and is actually a bio-degradable waste which is otherwise thrown off. Additionally the method of preparation is extremely simple and does not require skilled manual labour.

We have performed few experiments to access reusability of FBB for Cr (VI) adsorption. We found that, removal of Cr(VI) after adsorption is not possible from FBB surface under common laboratory condition. However development of protocol to desorb Cr(VI) from FBB are being carried out currently in our laboratory.

### Mechanism of Cr (VI) removal by FBB

**Figure 6:**
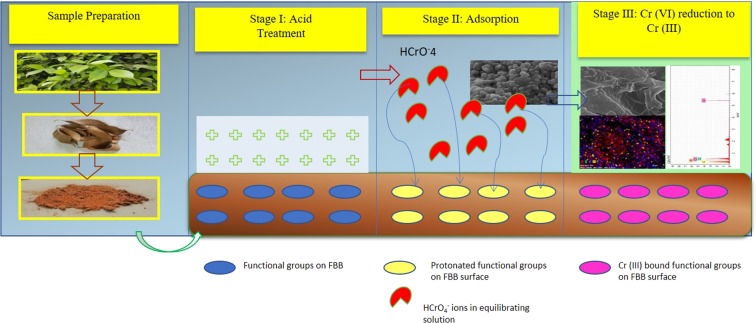
Proposed mechanism for Cr (VI) sorption on FBB and reduction of Cr (VI) to Cr (III) by functional groups present on FBB. The mechanism includes three stages: The first stage involves activation of the groups by acid treatment followed by the second stage in which adsorption of Cr (VI) takes place on FBB. The third stage involves the probable reduction of Cr (VI) to Cr (III).

It is known that sorption of oxyanions of Cr (VI) on the bio-adsorbents can be due to complexation with the ligands on surface, cation exchange, precipitation and/or electrostatic attraction between adsorbent and adsorbate^25^. In the present studies, complexation and precipitate formation is ruled out as any new compound formation is not observed. Hence at first glance, cation exchange and electrostatic attraction seem to be more feasible as the FBB surface was acid treated. In the present studies, preliminary experiments revealed that desorption of Cr (VI) from FBB surface is difficult indicating strong electrostatic attraction between FBB surface and Cr (VI). It is known that, bio-based adsorbents not only take up Cr (VI) in large quantities but also convert it to less harmful Cr (III) species. We propose that the uncharred FBB surface acquires positive charge due to protonation of basic functional groups after acid treatment. At lower pH values of equilibrating solutions, the dominant Cr (VI) species present negatively charged oxyionic HCrO_4_^−^. So it is obvious that when the two come in contact, HCrO_4_^−^ present in the solution gets adsorbed onto the positively charged FBB by electrostatic forces and then gets reduced to Cr (III) by carboxyl and amine groups present on FBB^26^. Proposed mechanism is described in Fig. 6.

## Conclusions

The present study has resulted into use of uncharred leaves of *Ficus benjamina* an effective adsorbent for Cr(VI) from aqueous solutions. Acid treatment enhanced the adsorption capacity of FBB. Kinetic studies revealed that the Adsorption of Cr(VI) onto biomaterial follows pseudo-second order kinetics and maximum adsorption takes place after an equilibration period of 24 hours. Thermodynamic studies showed that the process is thermodynamically favourable and endothermic in nature. Adsorption isotherm models clearly demonstrated that the Langmuir model was better fitting than the Freundlich model. Dubinin-Radushkevich model proved the sorption process to be physio-sorption. FTIR and FE-SEM-EDX studies show a marked difference in the FBB before and after Cr(VI) adsorption. The maximum amount of Cr (VI) adsorbed on FBB surface was found to be 56.85 mg g^−1^ which is better than many other biomaterials. The bio-adsorbent proved to be an effective, low cost alternative which can be employed by various industries generating Cr(VI) in waste water, where other sophisticated expensive methods are currently used.

## Material and Methods

### Materials

All the chemicals used in the present study were of analytical grade. Potassium dichromate (K_2_Cr_2_O_7_), hydrochloric acid (HCl), sulphuric acid (H_2_SO_4_), 1,5 diphenyl carbazide, sodium hydroxide (NaOH) and acetone were purchased from Loba Chemie (U.K) and used as received. All the stock aqueous solutions and working dilutions were prepared using deionized water (Milli-Q ultrapure water).

### Adsorbent preparation and acid treatment

Dry fallen leaves of *Ficus benjamina* were collected from MES Abasaheb Garware college campus in Pune, India. These leaves were washed thoroughly with distilled water for three to four times to remove dust and particulate matter from their surface. After washing leaves were sun dried, pulverized and sieved to obtain uniformity in the particle size. The pulverized leaves were then treated with 1 N HCl for 60 minutes on a shaker at 100 rpm to activate the surface functional groups. The temperature during shaking was 25.4 °C. The digest was repeatedly washed with distilled water to remove the excess acid. The digest was filtered, dried in shadow and stored in air tight containers till further use. This sample was designated as *Ficus benjamina* biomass (FBB). The schematic of the process is given in the Supplementary Fig. S1.

### Characterisation of FBB

The surface morphology of FBB was studied using field emission scanning electron microscope images (FE-SEM) using (FEI NOVANANO SEM-450) while energy dispersive X-ray measurements were carried out on Bruker energy dispersive X-Ray spectrometer (XFLASH-6I30). The functional group finger printing of FBB samples was carried out using FT-IR spectrometer (AMETEK STS-1900FT).

### Evaluation of optimal adsorption parameters

#### Batch adsorption experiments

To assess maximum uptake capacity of FBB, the experimental investigation was done in two steps, (i) determination of optimal conditions for uptake and (ii) kinetic and thermodynamic evaluation of the process. The percent removal of Cr (VI) from equilibrating solution and % uptake of Cr (VI) from equilibrating solution by given mass of FBB (q) was determined by using the Eqs. (15) and (16) respectively given below. The concentration of Cr (VI) was determined using a method previously developed in our laboratory^27^.

15 $$ \% Removal=\frac{({C}_{0}-{C}_{R})100}{{C}_{0}}$$

16 $${\rm{q}}=\frac{[{{\rm{C}}}_{0}-{{\rm{C}}}_{{\rm{e}}}]{\rm{V}}}{{\rm{M}}}$$

where, C_0_ (mg L^−1^) is the concentration of Cr (VI) in equilibrating solution and C_R_ is its concentration remaining in the equilibrating solution, q represents amount (mg) of Cr (VI) adsorbed per gram of the adsorbent, M is the mass of adsorbent (g) and C_0_ and C_e_ are initial and equilibrium concentrations (mg L^−1^) in the solution respectively and V is the volume of equilibrating solution in litres.

### Determination of optimum dose of FBB

Samples of FBB were weighed in the range of 0.01 to 0.20 g. They were added to a set of conical flasks each containing 20 mL of Cr (VI) solution of concentration 100 mg L^−1^. The mixtures were stirred on a magnetic stirrer at 100 rpm. The percentage of Cr (VI) removed from the solution and its amount taken up by FBB were determined using Eqs. (15) and (16) respectively.

### Determination of optimum pH and concentration of Cr (VI) solution

To determine the optimum pH of sorption 0.05 g portions of FBB were treated for 24 h with 20 mL aliquots of Cr (VI) solutions of 100 mg L^−1^. The pH of the solutions was adjusted using different buffers with pH values in the range of 1.00–8.00. The sorption capacity of FBB was determined using Eq. (16).

Investigation of optimum initial concentration of Cr (VI) in the equilibrating solution was done by equilibrating 0.05 g portions of FBB for a period of 24 h with 20 mL solutions containing Cr (VI) in the range of 20–200 mg L^−1^. The sorption capacities for each solution were determined using Eq. (16).

### Evaluation of kinetics of uptake by FBB

The equilibration time required for maximum sorption was determined by preparing a set of seven conical flasks, each containing 0.05 g FBB and 20 mL aliquots of 100 mg L^−1^ Cr (VI) over a period of 8 to 48 h. The amount of Cr (VI) adsorbed was measured periodically with intervals of time (8, 12, 16, 24, 28, 36 and 48 hours) and the removal percentage from the equilibrating solution was calculated using Eq. (15).

It was found that Cr (VI) sorption reaches a maxima in 24 hours and after that it almost remains constant. In order to understand the dynamics of the adsorption process, the uptake kinetics was studied. The study was performed at same conditions mentioned above except that the readings were taken after every one hour for a total time period of 24 hours. The observed data were fitted into pseudo-first and pseudo-second order kinetics^28,29^.

### Effect of temperature and thermodynamic studies

The uptake of Cr (VI) by FBB at different solution temperatures (25 to 80 °C) was studied by equilibrating 0.05 g of FBB for 24 h with 200 mg L^−1^ Cr (VI) solutions in a thermostat water bath (±0.1 °C). The sorption capacities were calculated using Eq. (16). To obtain the thermodynamic parameters, it was assumed that, the equilibrium between Cr (VI) in equilibrating solution and that is adsorbed on FBB is achieved in 24 hours of equilibration. The equilibrium constant thus obtained enabled to evaluate ΔG, ΔH and ΔS values for adsorption. The q values obtained by using Eq. (16) under optimized conditions were used to study the interactions among adsorbate and adsorbent using different adsorption isotherms.

All the experiments were performed in triplicates. The data and error bars in the figures indicate average ± SD.

## Supplementary information

Supplementary Figures and Tables
