# Supplementary material for: Valorization of Uncharred Dry Leaves of Ficus benjamina towards Cr (VI) removal from Water: Efficacy Influencing Factors and mechanism
Source: Sci Rep. 2019 Dec 18;9:19385. doi: 10.1038/s41598-019-55993-z (PMC6920481; doi:10.1038/s41598-019-55993-z)
Supplement: Supplementary file 1 — Supplementary Figures and Tables [file 41598_2019_55993_MOESM1_ESM.pdf]

Valorization of Uncharred Dry Leaves of *Ficus benjamina* towards Cr (VI) removal from Water: Efficacy Influencing Factors and mechanism

Preeti Kulkarni[1*], Varuna Watwe[1], Abubakar Hipparge[1], Sana Sayyad[1], Rutika Sonawane[1], Sunil Kulkarni[2],

[1]*Post-graduate and Research Centre, Department of Chemistry, MES Abasaheb Garware College, Pune, India*

[2]*Post-graduate and Research Centre, Department of Chemistry, Shikshana Prasarak Mandali's Sir Parashurambhau College, Tilak Road, Pune, India*

*Corresponding author

E-mail address: preeti.kulkarni@mesagc.org

Phone: +91 20 41038200

Figure S1. Collection and preparation of FBB

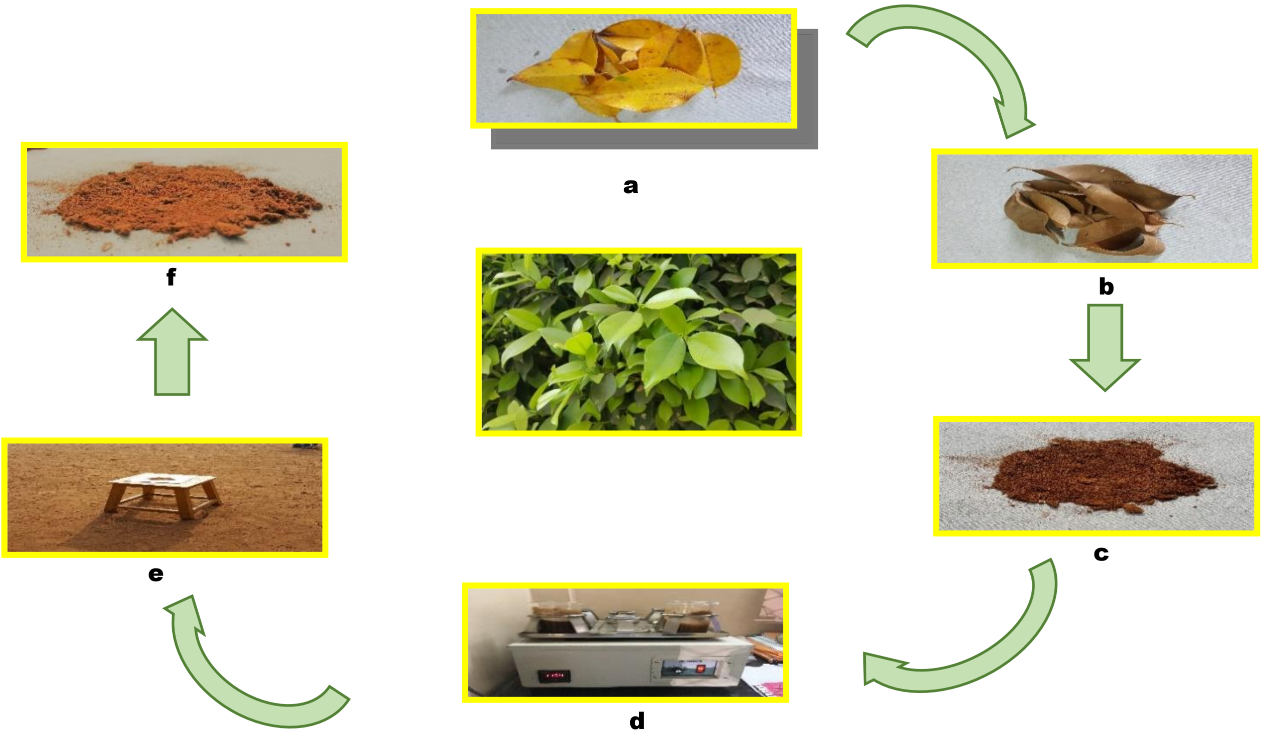

a) Dry fallen leaves of FB; b) Washed and sundried leaves of FB; c) pulverized and sieved FB leaves ; d) treatment of pulverized leaves with 1N HCl on shaker ; e) sun drying of acid treated pulverized leaves f) Ficusbenjaminabiowaste (FBB)

Figure S2: log R Vs log t

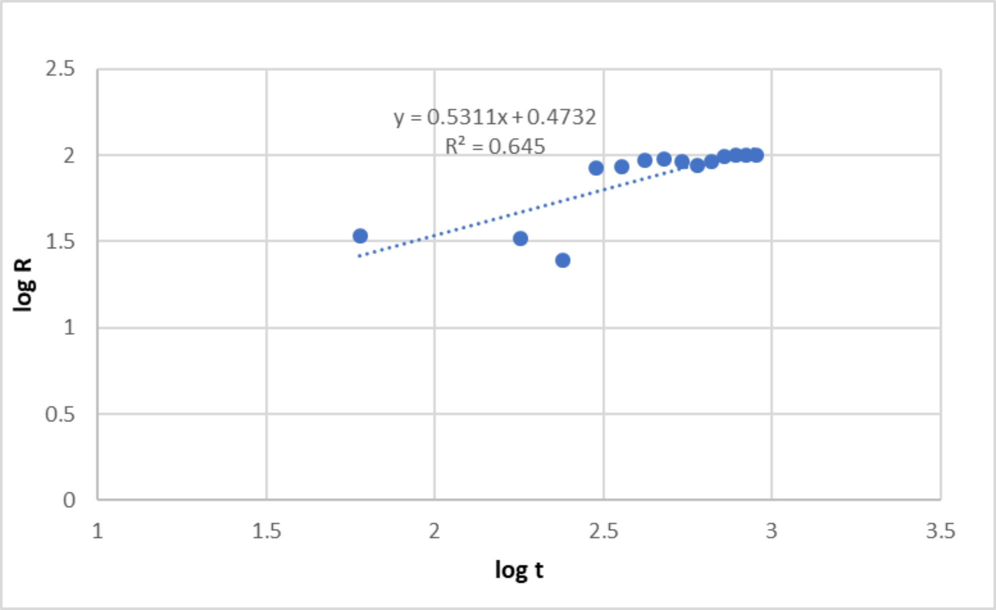

Experimental conditions – Initial concentration of Cr (VI); 80 ppm ; pH of Cr(VI) solution, 2 ; dose of FBB, 0.05 g ; temperature of Cr(VI) solution, 298 K ; volume of solution 20 ml.

Figure S3a: Pseudo-first order kinetics and **Figure S3b:** pseudo-second order kinetics

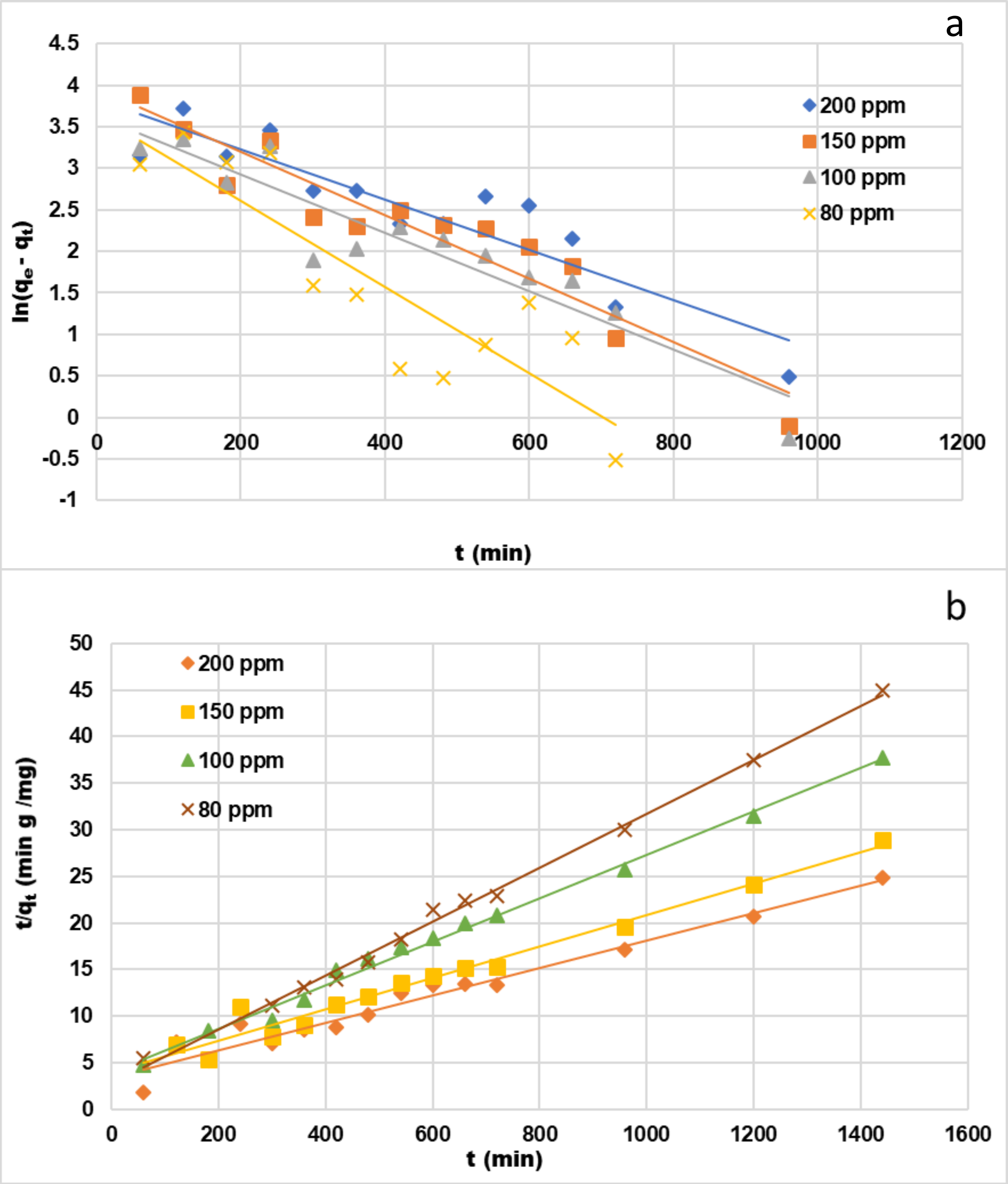

Figure S4:

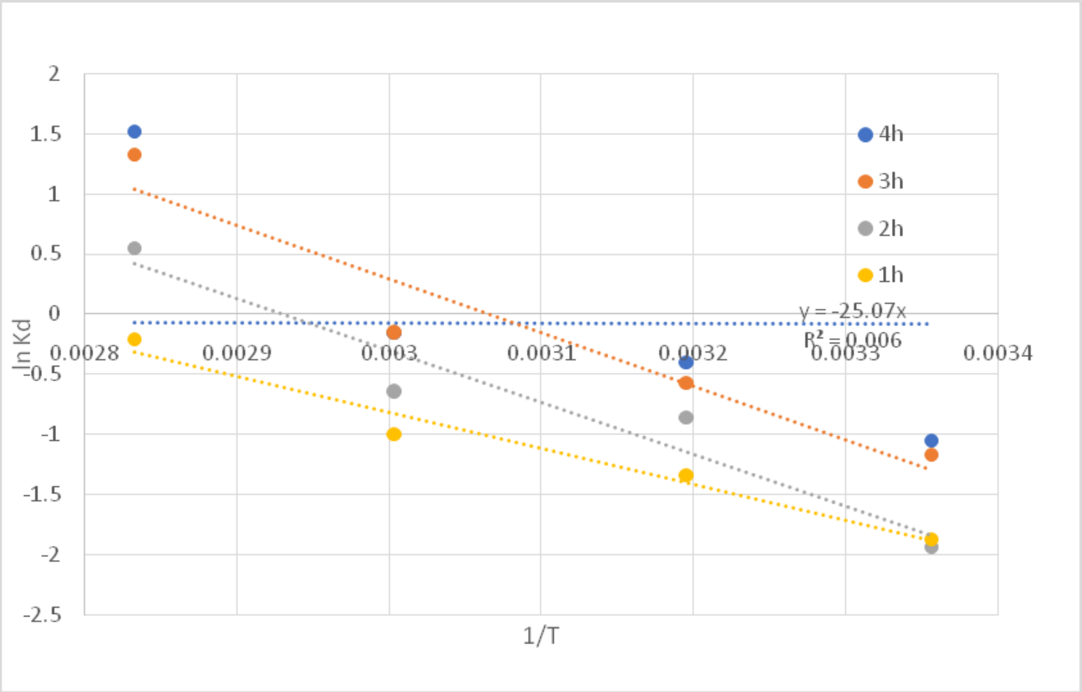
2
1.5
1
0.5
0
-0.5
-1
-1.5
-2
-2.5
0.0028
0.0029
0.003
0.0031
0.0032
0.0033
0.0034
ln Kd
1/T
4h
3h
2h
1h
y = -25.07x
R² = 0.006

Figure S5, S6, S7 and S8: Adsorption isotherms

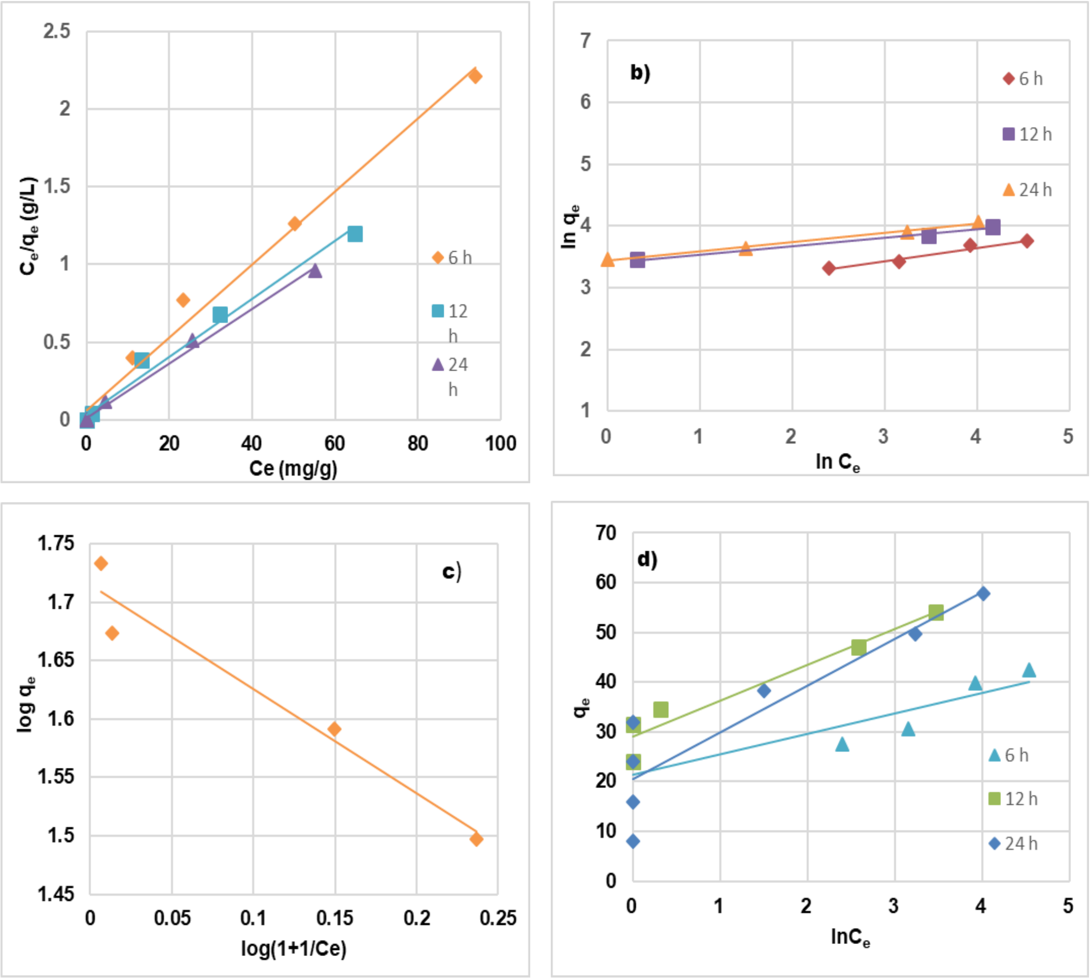

S5: Langmuir adsorption isotherm, S6: Freundlich adsorption isotherm, S7: Dubinin-Radushkevich adsorption isotherm and S8: Temkin adsorption isotherm

Table S1: Elemental composition of FBB by EDS analysis

a) FBB without Cr and b)FBB with Cr

a)

**El**	**AN**	**Series**	**unn. C [wt . %]**	**norm . C [wt . %]**	**Atom. C [at . %]**	**Error (1 Sigma) [wt . %]**
C	6	K-series	26.81	48.66	58.03	3.55
O	8	K-series	21.16	38.41	34.39	2.98
Si	14	K-series	4.44	8.07	4.11	0.22
N	7	K-series	1.43	2.59	2.65	0.51
Ca	20	K-series	1.26	2.28	0.82	0.09

b)

**El**	**AN**	**Series**	**unn. C [wt . %]**	**norm . C [wt . %]**	**Atom. C [at . %]**	**Error (1 Sigma) [wt . %]**
C	6	K-series	53.43	53.43	60.32	7.08
O	8	K-series	42.61	42.61	36.11	6.21
N	7	K-series	3.59	3.59	3.48	1.26
Cr	20	K-series	0.37	0.37	0.10	0.10
